# TaWAK2A-800, a Wall-Associated Kinase, Participates Positively in Resistance to Fusarium Head Blight and Sharp Eyespot in Wheat

**DOI:** 10.3390/ijms222111493

**Published:** 2021-10-25

**Authors:** Feilong Guo, Tianci Wu, Gangbiao Xu, Haijun Qi, Xiuliang Zhu, Zengyan Zhang

**Affiliations:** 1 The National Key Facility for Crop Gene Resources and Genetic Improvement, Institute of Crop Sciences, Chinese Academy of Agricultural Sciences, Beijing 100081, China; guofeilong1117@163.com (F.G.); wtc19961110@163.com (T.W.); haijunqi@yeah.net (H.Q.); zhuxiuliang@caas.cn (X.Z.); 2 The Laboratory of Forestry Genetics, College of Forestry, Central South University of Forestry and Technology, Changsha 410004, China; gangbiaoxu@163.com

**Keywords:** bread wheat (*Triticum aestivum*), chitin-induced immunity, *Fusarium graminearum*, *Rhizoctonia cereal*, wall-associated kinase (WAK)

## Abstract

Fusarium head blight (FHB) and sharp eyespot are important diseases of the cereal plants, including bread wheat (*Triticum aestivum*) and barley. Both diseases are predominately caused by the pathogenic fungi, *Fusarium graminearum* and *Rhizoctonia cerealis*. The roles of the wheat-wall-associated kinases (WAKs) in defense against both *F. graminearum* and *R. cerealis* have remained largely unknown. This research reports the identification of *TaWAK2A-800*, a wheat *WAK*-coding gene located on chromosome 2A, and its functional roles in wheat resistance responses to FHB and sharp eyespot. *TaWAK2A-800* transcript abundance was elevated by the early infection of *R. cerealis* and *F. graminearum*, or treatment with exogenous chitin. The gene transcript seemed to correspond to the resistance of wheat. Further functional analyses showed that silencing *TaWAK2A-800* compromised the resistance of wheat to both FHB (*F. graminearum*) and sharp eyespot (*R. cerealis*). Moreover, the silencing reduced the expression levels of six defense-related genes, including the chitin-triggering immune pathway-marker genes, *TaCERK1*, *TaRLCK1B*, and *TaMPK3*. Summarily, *TaWAK2A-800* participates positively in the resistance responses to both *F. graminearum* and *R. cerealis*, possibly through a chitin-induced pathway in wheat. *TaWAK2A-800* will be useful for breeding wheat varieties with resistance to both FHB and sharp eyespot.

## 1. Introduction

Bread wheat (*Triticum aestivum*) is one of the most important staple food crops in the world. However, wheat production is threatened in many areas by devastating pests and pathogens [1]. Fusarium head blight (FHB) in bread wheat or barley is caused by a complex of *Fusarium* fungi, which may be dominated by *Fusarium graminearum* [2]. FHB can lead to marked yield losses [3]. Moreover, cereal grains infected with the *Fusarium* species may contain high levels of mycotoxins, which threaten the health of human beings and animals [4]. *Rhizoctonia cerealis* is a necrotrophic fungal pathogen responsible for the sharp eyespot, which is a devastating disease of wheat and other cereal plants in some countries of the world, especially in China [5,6]. The breeding of disease-resistant wheat varieties is the most environmentally sustainable and effective strategy for minimizing the losses caused by FHB and sharp eyespot. Unfortunately, traditional resistance breeding is challenging since there are no wheat cultivars with full resistance to FHB or sharp eyespot [7,8]. To improve wheat resistance to *F. graminearum* and *R. cerealis*, it is important to isolate the pivotal resistance QTL/genes.

In plants, receptor-like kinases (RLKs) are involved in various life processes, such as growth, development, plant-pathogen interactions, and responses to environmental stimuli [9]. As a class of plant-specific RLKs, the protein sequences of wall-associated kinases (WAKs) and WAK-like (WAKLs) typically contain an aminol (N)-terminal signal peptide, an extracellular WAK-galacturonan-binding domain, an extracellular epidermal growth factor (EGF)-like domain, an EGF-calcium-binding (EGF-Ca^2+^) domain, a transmembrane domain, and a carboxyl (C)-terminal cytosolic serine/threonine kinase domain [10]. The extracellular EGF domain of WAKs has been determined to bind to the cell wall and persists during the process of plasmolysis [11]. Increasing evidence reveals that in several plant species, WAK/WAKL genes play an important role in plant-pathogen interactions. For instance, in *Arabidopsis thaliana,* the WAK gene, *AtWAKL22,* participates in resistance to *Fusarium oxysporm* [12]. In corn (*Zea mays*), *qHSR1* and *Htn1*, encoding two distinct *WAK* proteins, mediate the resistance of corn to head-smut disease and northern corn leaf blight, respectively [13,14]. In rice (*Oryza sativa*), the *WAK*-encoding gene, *Xa4’s*, high-expression improves resistance to bacterial blight disease [15]. In wheat, the *WAK*-encoding gene *Stb6*, isolated by the map-based cloning method, confers wheat resistance to *Septoria tritici* blotch disease caused by *Zymoseptoria tritici* [16]. Additionally, *TaWAK6*, another wheat WAK gene located on chromosome 5B, participates in adult plant resistance against wheat leaf rust [17]. Recently, Gadaleta et al. (2019) cloned an FHB-QTL based on map, located on the 2A chromosome, and introgressed from the Chinese wheat cultivar, Sumai 3, from durum wheat, and it corresponded to the sequence with TraesCS2A02G071800.1 (hereafter named *TaWAK2A-800*). The relative transcript level of the *TaWAK2A-800* gene in FHB-resistant wheat lines was significantly higher than in the susceptible wheat lines. After inoculation with *F. graminearum*, three TILLING mutant lines (Kronos3340, Kronos4209, Kronos3965) presented more severe FHB symptoms [18]. However, they did not demonstrate that *TaWAK2A-800* might have potential broad-spectrum resistance traits in wheat against other pathogens.

To explore if *TaWAK2A-800* participates in broad-spectrum resistance against both fungal pathogens, comparative transcriptomics and reverse transcription quantitative PCR (qRT-PCR) were used to test the *TaWAK2A-800* gene transcript profiles in the wheat response to *R. cerealis*. Moreover, we further dissected the functional role of *TaWAK2A-800* in wheat defense against *R. cerealis* and *F. graminearum* infection. After *R. cerealis* and *F. graminearum* infection, *TaWAK2A-800* was significantly induced in the *R. cerealis*-resistant wheat cultivar, CI12633, and the *F. graminearum*-resistant cultivar, Sumai 3. The functional assay results show that the expressed *TaWAK2A-800* is required not only for wheat resistance to both sharp eyespot and FHB, but also for the expression of the receptor-like kinase-coding gene, *TaCERK1*, the wheat receptor-like cytoplasmic kinase-coding *TaRLCK1B*, and the wheat mitogen-activated protein kinase-coding *TaMPK3*, *defensin*, *chitinase3*, and *β-1,3-glucanase* in wheat. This study reveals the function of *TaWAK2A-800* in the broad-spectrum resistance of wheat to both sharp eyespot by *R. cerealis,* and FHB by *F. graminearum*.

## 2. Results

### 2.1. Transcript Abundance of TaWAK2A-800 Seems to Link to Wheat Resistance Degree of R. cerealis and F. graminearum

**Figure 1:**
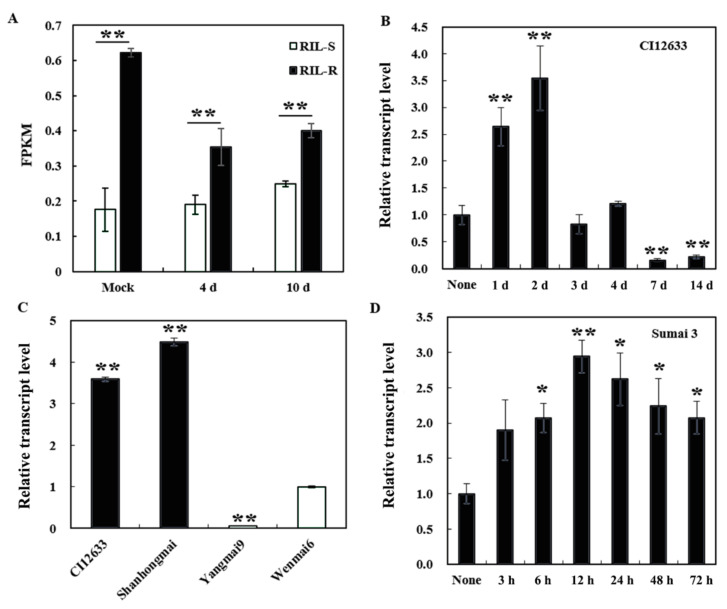
Transcriptional profiles of *TaWAK2A-800* in the wheat responses to *R. cerealis* and *F. graminearum*. (**A**) RNA-sequencing data of *TaWAK2A-800* transcription between RIL-R and RIL-S. (**B**) qRT-PCR analysis of *TaWAK2A-800* in the resistant wheat cultivar, CI12633, inoculated with the pathogen, *R. cerealis*. (**C**) qRT-PCR analysis of *TaWAK2A-800* in four wheat cultivars with different degrees of sharp eyespot resistance at 2 dpi with *R. cerealis*. The transcriptional level of *TaWAK2A-800* in the Wenmai 6 was set to 1. (**D**) qRT-PCR analysis of *TaWAK2A-800* in the resistant wheat cultivar, Sumai 3, plants inoculated with *F. graminearum*. The transcriptional level of *TaWAK2A-800* without treatment (none) is set to 1. Student’s *t*-test (* *p* < 0.05; ** *p* < 0.01). Bars indicate standard error of the mean from three biological replications. The wheat actin gene was used as an internal control.

On the basis of the RNA-sequencing data from sharp-eyespot-resistant and -susceptible recombinant inbred lines (RILs, cross of Shanhongmai and Wenmai 6), inoculation with the *R. cerealis* strain, RC207, at 4 and 10 days, and also mock, the gene, *TaWAK2A-800* (sequence with ID *TraesCS2A02G071800.1*), was significantly higher in the resistant RIL pool (RIL-R) than in the susceptible RIL pool (RIL-S) at the same inoculation time points (Figure 1A).

Moreover, we examined the relative transcript level of *TaWAK2A-800* in the wheat response to the pathogen, *R. cerealis*, by qRT-PCR. The analyses showed that the *TaWAK2A-800* gene transcript was rapidly induced in the sharp-eyespot-resistant wheat cultivar, CI12633, at day 1, post inoculation (dpi) and 2 dpi, and reached a peak at 2 dpi (Figure 1B). Interestingly, at 2 dpi, with *R. cerealis* compared with the sharp-eyespot-susceptible cultivars, Yangmai 9 and Wenmai 6, *TaWAK2A-800* transcript abundance was significantly higher in the sharp-eyespot-resistant wheat cultivars (CI12633 and Shanhongmai) (Figure 1C). This suggests that the transcript abundance of *TaWAK2A-800* might correspond to the resistance degree of the wheat cultivars. In addition, the transcript of *TaWAK2A-800* was significantly induced upon *F. graminearum* inoculation in the *FHB*-resistant wheat cultivar, Sumai 3 (Figure 1D), similar to the expression patterns of two FHB-resistance genes studied in previous papers [18,19]. The above results suggests that *TaWAK2A-800* was involved in the wheat defense responses, not only against *R. cerealis*, but also against *F. graminearum*.

### 2.2. Sequence Characterization of TaWAK2A-800 in Wheat

**Figure 2:**
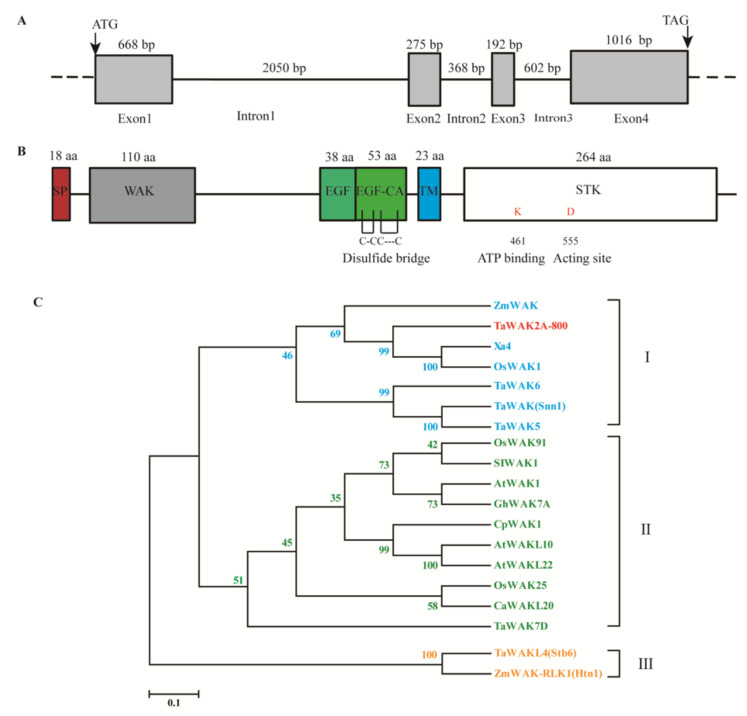
Sequence and phylogenetic analyses of *TaWAK2A-800*. (**A**) Genomic structure of the *TaWAK2A-800* gene. (**B**) Schematic diagram of the domain (shaded area) of the TaWAK2A-800 protein. The red box represents a signal peptide, the gray box represents the WAK-GUB domain, the green boxes represent EGF domains, the blue box represents the transmembrane domain, and the white box represents the STK domain. (**C**) Phylogenetic analysis of TaWAK2A-800 protein and 18 other WAK/WAKL proteins. The bootstrapped phylogenetic tree is constructed by using the neighbor-joining phylogeny of MEGA 7.0.

The *TaWAK2A-800* genomic sequence was cloned from DNA taken from the stems of the wheat cultivar, Wenmai 6. According to a BLAST analysis against the hexaploid wheat genome sequence, the full-length cDNA coding sequence of *TaWAK2A-800* contains a complete open reading frame (ORF) with 2151 bp, and the gene locates on the short arm of wheat chromosome 2A. A comparison of the cDNA and genomic sequences indicates that the *TaWAK2A-800* genomic sequence contains four exons and three introns (Figure 2A). The deduced protein, TaWAK2A-800, was comprised of 716 amino acid (aa) residues, its molecular weight was 77.88 kD, and the theoretical isoelectric point (pI) was 6.65. There was a predicted signal peptide (at no. 1–18 amino acids, aa) in the N-terminal region, an EGF-like domain (no. 277–314 aa), and EGF-CA (no. 315–367 aa), with four conserved cysteine residues. Moreover, the C-terminal of the predicted TaWAK2A-800 harbored a cytosolic STK domain (no. 434–697 aa), which included an ATP binding site and an acting site (Figure 2B). All the structural characteristics of the predicted TaWAK2A-800 protein were coincident with those of the reported WAK proteins. Thus, TaWAK2A-800 might be a putative WAK protein. Because bread wheat is hexaploid and contains A, B, and D sets of genomes, three copies of *TaWAK2A-800* were expected. In fact, after BLAST in the chromosome-based draft sequence of the hexaploid wheat using *TaWAK2A-800*, only one homoeologous gene from the wheat cultivar Chinese Spring was found on the wheat chromosome 2D, named as *TaWAK-2D-600* (*TraesCS2D02G070600.1*), with a 2139 bp ORF. A pairwise comparison showed that the ORF and the amino acid sequences of *TaWAK2A-800* shared 93.18% and 89.42% identities, respectively, with those of *TaWAK-2D-600*.

Phylogenetic analysis was performed to decipher the relationship among TaWAK2A-800 and the other 18 related WAKs/WAKLs from wheat, rice, maize, *A. thaliana*, cotton (*Gossypium hirsutum*), *Craterostigma plantagineum*, *Capsicum annuum*, and *Solanum lycopersicum*. The phylogenetic tree indicated that TaWAK2A-800 with Xa4 and OsWAK1 from rice, TaWAK5, TaWAK6, and TaWAK (Snn1) from wheat, and ZmWAK (encoded by *qHSR1*) from maize, were clustered on the same clade. Other WAKs were clustered into the second clade, including GhWAK7A from cotton, AtWAK1, AtWAKL10 and AtWAKL22 from *A. thaliana*, OsWAK91 and OsWAK25 from rice, CaWAKL20 from *Capsicum annuum*, CpWAK1 from *C. plantagineum*, TaWAK7D from wheat, and SIWAK1 from *Solanum lycopersicum*. Meanwhile, TaWAKL4 (Stb6) and ZmWAK-RLK (Htn1) were clustered into the third clade (Figure 2C). Moreover, we further compared the protein sequences of TaWAK2A-800 and five reported wheat WAKs to determine their identity. TaWAK2A-800 shared 35.63%, 35.52%, 31.08%, 34.71%, and 18.44% protein sequence identities with five wheat known-functional WAKs, namely, TaWAK5, TaWAK6, TaWAK7D, TaWAK (Snn1), and TaWAKL4 (Stb6), respectively. The above results indicate that TaWAK2A-800 is a WAK member that is distinct from the five reported wheat WAKs.

### 2.3. Knocking-Down TaWAK2A-800 Compromises Wheat Resistance to Fusarium Head Blight and Sharp Eyespot

To investigate whether the role of *TaWAK2A-800* in wheat resistance to *F. graminearum*, *TaWAK2A-800* was knocked-down (silenced) in the resistant wheat cultivar, Sumai 3, via the barley yellow dwarf virus (BSMV)-mediated VIGS method, and then the disease severities of these plants were assayed. Briefly, Si-Fi software prediction showed that the 172 bp fragment specific to *TaWAK2A-800* (Table 1) was screened to be a VIGS fragment, and then subcloned in the multiclone site of the BSMV γ chain in order to generate the BSMV:TaWAK2A-800 recombinant construct.

**Figure 3:**
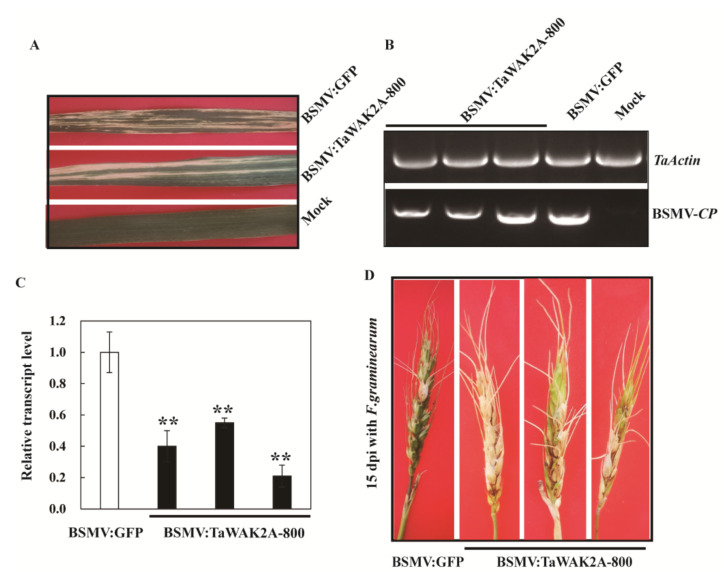
Silencing of *TaWAK2A-800* by BSMV-induced gene silencing increases Sumai 3 susceptibility to *F. graminearum*. (**A**) Mild chlorotic mosaic symptom displayed on the newly emerged leaves at 15 dpi with BSMV:GFP or BSMV:TaWAK2A-800. (**B**) RT-PCR analysis of the transcription of BSMV *CP* in the wheat plants transfected by BSMV:GFP or BSMV:TaWAK2A-800 at 15 days. (**C**) qRT-PCR analysis of the *TaWAK2A-800* transcript abundance in the Sumai 3 wheat plants transfected by BSMV:GFP or BSMV:TaWAK2A-800 at 15 dpi. The transcript level of *TaWAK2A-800* in BSMV:GFP-transfected Sumai 3 is set to 1. Student’s *t*-test (** *p* < 0.01). Bars indicate standard error of the mean from three biological replications. Three replicates were averaged and statistically treated (*t*-test: ** *p* < 0.01). Bars indicated standard error of the mean. (**D**) FHB symptoms of the control and TaWAK2A-800-silenced Sumai 3 spikes at 20 dpi with *F. graminearum*.

After transfection with BSMV: TaWAK2A-800, or BSMV:GFP viruses, for 10 days, the leaves of the transinfected wheat Sumai 3 plants showed mild chlorotic mosaic symptom of BSMV (Figure 3A). RT-PCR analysis showed that the transcription of BSMV coat protein (*CP*) in the wheat plants transfected by BSMV:GFP, or BSMV:TaWAK2A-800, at 15 days could be detected (Figure 3B), proving that these Sumai 3 plants were successfully infected by BSMV: TaWAK2A-800, or BSMV:GFP viruses. Further, the qRT-PCR analysis suggested that, compared with the control (BSMV: GFP transfected Sumai3) plants, the expression of *TaWAK2A-800* was markedly downregulated in the BSMV: TaWAK2A-800-infected Sumai 3 plants (Figure 3C). To assess the resistance function of *TaWAK2A-800* to FHB, the inoculum of the *F. graminearum* strain, F0609 (100 spores/μL), were injected into the spikelets of the spikes of the BSMV-transfected Sumai 3 using a syringe. At 20 dpi with *F. graminearum*, the FHB symptoms in the *TaWAK2A-800*-silenced Sumai 3 plants were more severe than in BSMV: GFP-transfected Sumai 3 (control) plants (Figure 3D). Accordingly, in two batches, the average number of scabbed spikelets, and the average percentage of the scabbed spikelets of the *TaWAK2A-800*-silenced Sumai 3 plants, were 3.85 and 4.36, 36.99% and 39.87%, respectively. Meanwhile, the average number of scabbed spikelets, and the average percentage of the scabbed spikelets of BSMV:GFP-treated Sumai 3 plants, were 2.76 and 3.18, 26.67% and 28.23%, respectively. The results suggest that the knocking-down of *TaWAK2A-800* in Sumai 3 compromised the resistance of the wheat cv. Sumai 3 to FHB caused by *F. graminearum*, suggesting that *TaWAK2A-800* expression is required for the resistance of the *F. graminearum*-resistant wheat cv. Sumai 3 to FHB caused by *F. graminearum*.

**Figure 4:**
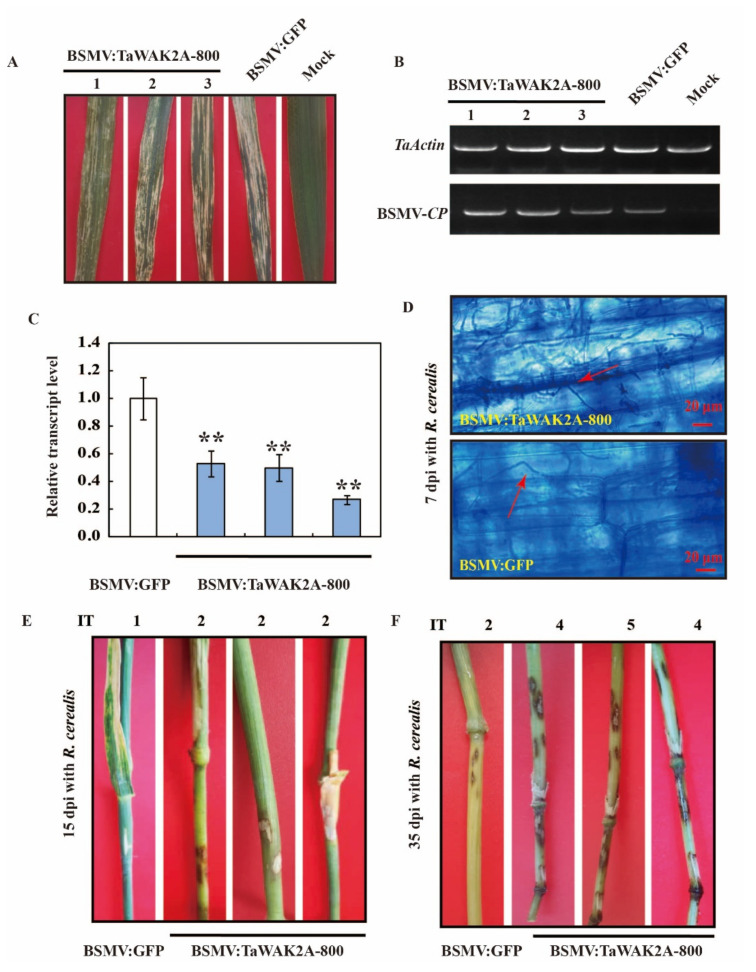
Knocking-down of *TaWAK2A-800* by BSMV-induced gene silencing impairs resistance of the wheat cv. CI12633 to the pathogen *R. cerealis*. (**A**) Mild chlorotic mosaic symptoms displayed on newly emerged leaves at 15 dpi with BSMV:GFP, or BSMV: TaWAK2A-800, transfection. (**B**) RT-PCR analysis of the transcription of BSMV *CP* in the wheat plants transfected by BSMV:GFP, or BSMV:TaWAK2A-800, for 15 d. (**C**) qRT-PCR analysis of the transcript abundance of *TaWAK2A-800* in the CI12633 wheat plants transfected by BSMV:GFP, or BSMV:TaWAK2A-800, for 15 d. The transcript level of *TaWAK2A-800* in BSMV:GFP-transfected CI12633 is set to 1. Student’s *t*-test (** *p* < 0.01). (**D**) The *R. cerealis* hyphae on the base leaf sheath of the BSMV: TaWAK2A-800- and BSMV:GFP-transfected CI12633 plants at 7 dpi with *R. cerealis,* WK207, were stained with trypan blue. Bar = 20 μm. (**E**) Wheat sharp eyespot symptoms of BSMV:GFP- and BSMV:TaWAK2A-800-transfected CI12633 stems at 15 dpi with *R. cerealis*. (**F**) Wheat sharp eyespot symptoms on BSMV:GFP-transfected and BSMV:TaWAK2A-800-silenced CI12633 stems at 35 dpi with *R. cerealis* WK207.

To examine whether *TaWAK2A-800* expression is also required for the innate immunity of the wheat plant to *R. cerealis*, *TaWAK2A-800* transcript was knocked-down by VIGS in the *R. cerealis*-resistant wheat cv. CI12633. Fifteen days post the virus transfection into the wheat leaves, the mild chlorotic mosaic symptoms and *CP* of the BSMV transfection could be observed on newly emerged leaves (Figure 4A) and was readily detected in the virus-transfected leaves, but not in the mock plants (Figure 4B), indicating that the BSMV-treated wheat plants were successfully transfected by BSMV. Compared with BSMV: GFP-transfected CI12633 plants, the *TaWAK2A-800* transcription was substantially knocked-down/silenced in BSMV:TaWAK2A-800-transfected CI12633 plants (Figure 4C). Furthermore, the BSMV: GFP-transfected, and the BSMV: TaWAK2A-800-silenced, CI12633 plants were inoculated with the pathogenic *R. cerealis* strain, WK207. Microscopic observation showed that, at 7 dpi with WK207, the *R. cerealis* hyphae density on the inoculated base sheaths of the TaWAK2A-800-silenced wheat plants was more than that on the BSMV:GFP-transfected wheat plants (Figure 4D), providing evidence that silencing *TaWAK2A-800* impaired the resistance of CI12633 against the hyphae development of *R. cerealis*. At 15 dpi, with the pathogen *R. cerealis*, brown lesions (the typical symptoms of sharp eyespot) displayed on the sheaths and stems of the TaWAK2A-800-silenced CI12633 plants but displayed, to a lesser extent, on the sheaths and stems of the BSMV: GFP-treated (control) CI12633 (Figure 4E). Moreover, with the pathogen *R. cerealis*, at 35 dpi, the stems of the TaWAK2A-800-silenced CI12633 plants presented more serious necrotic areas than the BSMV: GFP-infected CI12633 plants (Figure 4E). In two batches of VIGS and disease scoring at 35 dpi with the pathogen, *R. cerealis* WK207, the average infection types (ITs) on the stems of the TaWAK2A-800-silenced wheat cultivar CI12633 plants were between 2.50 and 2.89 (Figure 4F), while those of the BSMV:GFP-infected CI12633 (control) plants were 1.71 to 1.93, respectively. These results indicate that knocking-down *TaWAK2A-800* in the wheat cultivar, CI12633, could cause higher susceptibility to the pathogen *R. cerealis*, and suggests that *TaWAK2A-800* was required for wheat-resistance responses against pathogenic *R. cerealis* infection.

### 2.4. TaWAK2A-800 May Contribute to Chitin-Induced Defense Pathway

**Figure 5:**
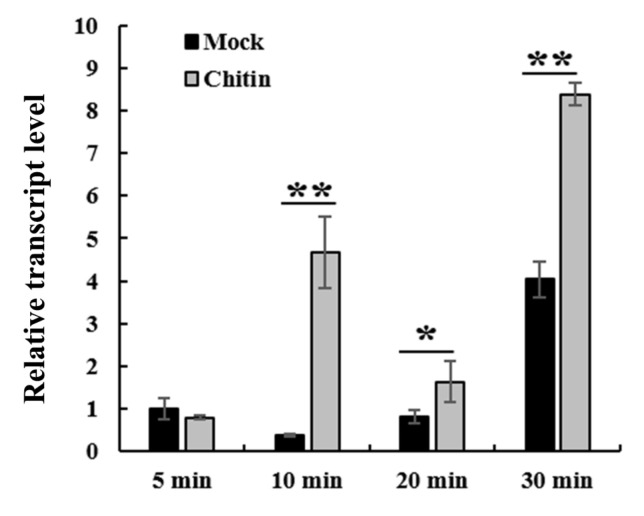
*TaWAK2A-800* contributed to chitin-induced immune pathway. Expression patterns of *TaWAK2A-800* in wheat cultivar CI12633 plants after treatment with exogenous chitin for 5, 10, 20, and 30 min. Student’s *t*-test (* *p* < 0.05; ** *p* < 0.01). Bars indicate standard error of the mean from three biological replications. *TaActin* was used as an internal control.

As a conserved component of the fungal cell wall, chitin can trigger plant immune response [20]. To investigate how *TaWAK2A-800* responds to exogenous chitin stimuli, we analyzed the transcript profiles of *TaWAK2A-800* in wheat cv. CI12633 treated with 100 μg/mL chitin [21], or with mock solution, for 5, 10, 20, and 30 min. The analyses showed that the *TaWAK2A-800* transcript level was significantly elevated by chitin treatment compared with the mock treatment (Figure 5).

### 2.5. Silencing TaWAK2A-800 Decreases the Transcripts of Defense-Related Genes in Wheat

**Figure 6:**
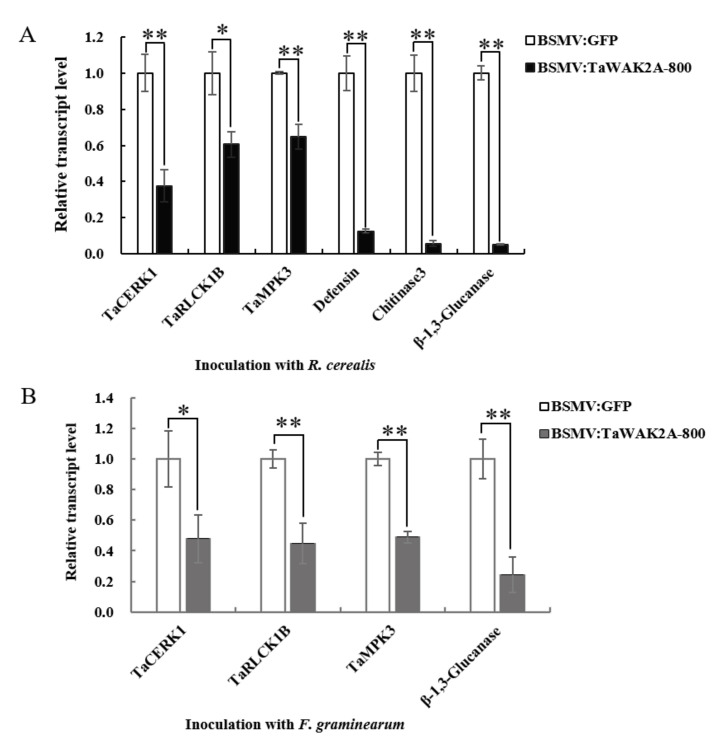
Transcription profiles of defense-associated genes *TaCERK1*, *TaRLCK1B*, *TaMPK3*, *Defensin*, *Chitinase3*, and *β-1,3-Glucanase* in BSMV: GFP-transfected, and BSMV:TaWAK2A-800-silenced, wheat plants inoculated with (**A**) *R. cerealis* and (**B**) *F. graminearum*. Statistically significant differences between BSMV: GFP-transfected and BSMV:TaWAK2A-800-silenced wheat plants were determined using Student’s *t*-test (* *p* < 0.05; ** *p* < 0.01). Bars indicate standard error of the mean from three biological replications. *TaActin* was used as an internal control.

To investigate if the silencing of *TaWAK2A-800* represses the expression of defense-associated genes in wheat, qRT-PCR was deployed to examine the transcription levels of wheat defense-associated genes in *TaWAK2A-800*-silenced and BSMV:GFP-transfected wheat plants inoculated with the pathogens *R. cerealis,* or *F. graminearum*. As is shown in Figure 6A,B, after inoculation with *R. cerealis* and *F. graminearum*, the expression levels of the wheat receptor-like kinase-coding gene, *TaCERK1*, the wheat receptor-like cytoplasmic kinase, *TaRLCK1B*, and the wheat mitogen-activated protein kinase, *TaMPK3,* were decreased in the BSMV:TaWAK2A-800-infected wheat plants compared with the BSMV:GFP-transfected wheat plants. Moreover, compared with the BSMV:GFP-transfected (control) wheat plants, the transcript levels of three wheat defense-marker genes, including *defensin*, *chitinase3*, and *β-1,3-glucanase*, were significantly reduced in *TaWAK2A-800*-silenced wheat plants after *R. cerealis* inoculation (Figure 6A). In addition, after inoculation with *F. graminearum*, only *β-1,3-Glucanase* expression was significantly downregulated in *TaWAK2A-800*-silenced Sumai 3 wheat plants compared with the BSMV:GFP-infected (control) plants (Figure 6B). These data suggest that expressed *TaWAK2A-800* is also required for the expression of these defense-associated genes.

## 3. Discussion

In Arabidopsis, rice, maize, cotton, and wheat, some WAK/WAKL proteins were reported to have an important function in resistance responses to the bacterial or fungal pathogens [14,15,16,17,21,22,23,24,25,26]. In this study, on the basis of the RNA-seq data and qRT-PCR analyses, we identified the wheat-wall-associated kinase gene, *TaWAK2A-800,* with broad-spectrum resistance against *R. cerealis* and *F. graminearum*. *TaWAK2A-800* belongs to the WAK subfamily, but its protein sequence is distinct from those of the five reported wheat WAKs, including TaWAK5, TaWAK6, TaWAK7D, TaWAK (Snn1), and TaWAKL4 (Stb6). The deduced protein sequence of TaWAK2A-800 contains one signal peptide, one galacturonan-binding GUB domain, one EGF-like domain, one EGF-CA domain, one transmembrane region, and a non-RD kinase domain. Among various types of RLKs, non-RD RLKs predominantly bind to native immune receptors, recognize conserved microbial characteristics, activate PTI, and even confer disease resistance [27]. For instance, four reported WAK proteins, including ZmWAK-RLK1 [13], ZmWAK [14], Xa4 [15], and TaWAK6 [17], all contain a non-RD kinase domain that mediates disease resistance. Our functional analyses revealed that TaWAK2A-800 also belongs to a non-RD-type WAK protein in wheat, and positively participates in the resistance responses of wheat to sharp eyespot and FHB.

Further, qRT-PCR analyses showed that, compared with nontreatment, the expression level of *TaWAK2A-800* in wheat was significantly upregulated by *F. graminearum* infection, or in the early infection stage (1–2d), of the pathogen *R. cerealis*. Intriguingly, after inoculation with *R. cerealis* for 2 d, the transcript level of *TaWAK2A-800* was significantly higher in *R. cerealis*-resistant wheat cultivars, Shanhongmai and CI12633, than in the susceptible wheat cultivars, Yangmai 9 and Wenmai 6. Importantly, knocking-down *TaWAK2A-800* impaired the resistance of wheat to both sharp eyespot caused by *R. cerealis* infection, and FHB caused by *F. graminearum* infection. A previous study reported that *TaWAK2A-800* was responsible for FHB-resistance QTL in wheat [18]. Herein, our functional dissection results were not only in line with the aforementioned reported conclusion, but also revealed a new defensive role for *TaWAK2A-800* in the wheat immune response to sharp eyespot. Further, the current study reveals a potentially conserved mechanism of wheat resistance against *F. graminearum* and *R. cerealis*.

Previous studies have shown that three types of *PR*-encoding genes, including *Defensin*, *Chitinases*, and *β-1,3-Glucanase*, contribute to the resistance of wheat to sharp eyespot [28,29]. To understand the potential molecular mechanisms of *TaWAK2A-800* in the defense response against both pathogens, we examined the transcripts of *Defensin*, *Chitinase 3*, and *β-1,3-Glucanase* in BSMV: TaWAK2A-800-silenced wheat, and the BSMV:GFP-transfected wheat plants, inoculated with *R. cerealis* or *F. graminearum*. The results show that, compared with BSMV:GFP-transfected wheat plants, the transcript levels of the wheat, *Defensin*, *Chitinase 3*, and *β-1,3-Glucanase* were lower in the more susceptible *TaWAK2A-800*-silenced wheat plants after inoculation with *R. cerealis* or *F. graminearum*. The data suggest that TaWAK2A-800 might indirectly activate the expression of the above defense-marker genes in wheat resistance responses against *R. cerealis* or *F. graminearum*.

Chitin, a typical pathogen-associated molecular pattern (PAMP) on the fungal cell wall, can trigger the plant innate immune response by activating PAMP-triggered immunity (PTI) in many plant species [30]. In Arabidopsis and rice, the receptor complex, composed of LYK5 and CERK1 (an RLK with an extracellular lysine motif domain), can recognize fungal chitin [31,32,33]. Upon chitin perception, CERK1 phosphorylates the RLCK PBL27 in Arabidopsis [34]. In the knockout mutation of *PBL27*, the chitin-dependent activation of the MAPKs (MPK3 and MPK6) was markedly reduced [35]. In cotton, the wall-associated kinase, GhWAK7A, interacts with the chitin sensory receptors, GhCERK1 and GhLYK5, to phosphorylate GhLYK5, activating the chitin-induced defense responses to *Verticillium dahliae* and *Fusarium oxysporum* f.sp. *vasinfectum* [21]. In this study, the *TaWAK2A-800* transcript level was significantly induced by chitin treatment. Moreover, compared to the control wheat plants, the transcript abundance of the chitin-induced immune pathway marker genes, *TaCERK1*, *TaRLCK1B*, and *TaMPK3,* were reduced in *TaWAK2A-800*-silenced wheat plants after inoculation with *R. cerealis* and *F. graminearum*. These results suggest that *TaWAK2A-800* contributes to the wheat defense responses to *R. cerealis* and *F. graminearum,* possibly through a chitin-induced defense pathway. Similarly, the transcripts of rice *OsWAK90/91* were upregulated after chitin treatment, and boosted resistance against rice blast caused by *Magnaporthe oryzae* [22].

## 4. Materials and Methods

### 4.1. Plants Material, Fungal Isolates, and Primers

Four wheat cultivars/varieties, including *R. cerealis*-resistant CI12633 (Prof. Shibing Cai, Jiangsu Academy of Agricultural Sciences, Nanjing, Jiangsu, China), and Shanhongmai (Prof. Jizeng Jia, Institute of Crop Sciences, CAAS, Beijing, China), *R. cerealis*-susceptible Yangmai 9 (Prof. Jizeng Jia), and Wenmai 6 (Prof. Shunhe Cheng, Jiangsu Academy of Agricultural Sciences, Nanjing, Jiangsu, China) [36], were used to investigate the transcriptional profile of *TaWAK2A-800*. The sharp-eyespot-resistant and -susceptible recombinant RILs (derived from Shanhongmai and Wenmai 6 cross) were used for RNA-sequencing, and RNA-sequencing analyses were performed as described by Guo et al. [37]. The *R. cerealis*-resistant cultivar, CI12633, and the *F. graminearum*-resistant cultivar, Sumai 3 (Prof. Jizhong wu, Jiangsu Academy of Agricultural Sciences, Nanjing, Jiangsu, China), were used to detect the defense role of *TaWAK2A-800* against *R. cerealis* and *F. graminearum* by the virus-induced gene-silencing (VIGS) experiment, respectively. The fungal pathogenic *R. cerealis* isolate, WK207, and the *F. graminearum* isolate, F0609, were provided by Prof. Jinfeng Yu (Shandong Agricultural University, Tai’an, Shandong, China) and Prof. Miaoping Zhou (Jiangsu Academy of Agricultural Sciences, Nanjing, Jiangsu, China), respectively.

All wheat plants were grown in a glasshouse in 14-h light/10-h dark (22 °C/12 °C) conditions. At the tillering growth stage, these wheat plants (CI12633, Shanhongmai, Yangmai 9, Wenmai 6) were further inoculated on each stem base with small toothpicks harboring the well-developed mycelia of *R. cerealis*. Then, the wheat plants were grown in a growth chamber at 22 °C and high humidity during the first 14 days. To prepare conidial suspensions for inoculation, *F. graminearum* mycelia were collected from potato agar plates, then transferred into liquid mung bean medium, and cultivated at 25 °C for 3 d with shaking at 150 rpm. The conidial suspension was filtered, centrifuged, resuspended, and adjusted to 100/μL in sterile water. At the anthesis of Sumai 3, the suspension was further injected into a spikelet of spike using a syringe. Then, these spikes of Sumai 3 were placed in a plastic moist chamber, with high humidity, at 22 °C for 2 days. The RNA-sequencing-based transcriptomics of *TaWAK2A-800* (Table S1) were subjected as described by Guo et al. [37].

Chitin was suspended in sterile water and used at a final concentration of 100 μg/mL [24]. Wheat seedlings were sprayed with distilled water (mock), or chitin solution. At 5, 10, 20, and 30 min, we collected the wheat leaves corresponding to each treatment, and extracted RNAs for RT-qPCR analysis.

### 4.2. Cloning and Sequence Analysis of TaWAK2A-800

Total plant RNA extraction was performed following Zhang et al. [38]. This *TaWAK2A-800* gene sequence was cloned from the wheat cultivar Wenmai 6 leaves based on the means of two rounds of nest PCR reactions by gene-specific primer (*TaWAK2A-800*-F1: 5′-CAACGCCACACTATCCAGGT-3′, *TaWAK2A-800*-R1: 5′-TGTTCCC TTCCCACCTCTAGT-3′; *TaWAK2A-800*-F2: 5′-CATCAGGTCGACACAGCAGG-3′, *TaWAK2A-800*-R2: 5′-CAGCCGGGAGATTAGGAAGC-3′) following the above sequence. TraesCS2A02G071800.1 was downloaded from the Chinese Spring V1.0 genome (http://202.194.139.32/jbrowse.html, accessed on 27 February 2020). The deduced protein sequence of TaWAK2A-800 and the phylogenetic tree were obtained and constructed as described by Guo et al. [37].

### 4.3. Assay for the Function of TaWAK2A-800 in Wheat Defense against FHB and Sharp Eyespot

The VIGS fragment (172 bp) of *TaWAK2A-800,* and its specificity and efficient siRNA hits, were predicted by the Si-Fi software (http://labtools.ipk-gatersleben.de/, accessed on 24 April 2020). The specific fragment of *TaWAK2A-800* was amplified from the cDNA sequence for *TaWAK2A-800* using a gene-specific primer (*TaWAK2A-800*-γ-F: 5′-CTAGCTAGCGCCTACCATGGGAGAAGTCAG-3′, *TaWAK2A-800*-γ-R: 5′-CTAGCTAGCACATCACACACTGGAGAAAACA-3′) from the CI12633, and inserted in an antisense orientation into the site of the RNAγ of the part of the BSMV to generate a BSMV: *TaWAK2A-800* construct. Following Zhu et al. [36], the mixtures of the transcribed RNAs of the tripartite DNA chains of BSMV: TaWAK2A-800, and BSMV: GFP (as control), were separately transfected. At least 20 CI12633 or Sumai 3 wheat plants were transfected at the three-leaf stage, and then moisturized for at least 2 d. The wheat tissue samples were harvested and analyzed to monitor BSMV infection at 15 d after virus inoculation by a gene-specific primer (BSMV-CP-F: 5′-TGACTGCTAAGGGTGGAGGA-3′, BSMV-CP-R: 5′-CGGTTGAACATCACGAA GAGT-3′). Following the previous method [29,39], the BSMV-transfected Sumai 3 or CI12633plants were inoculated with the *F. graminearum* isolate, F0609, or the *R. cerealis* isolate, WK207, at 15 days transfected with BSMV, and then scored for disease symptoms at 20 and 35 days post the fungal inoculation.

### 4.4. RT-PCR and qRT-PCR

The transcriptional levels of *TaWAK2A-800* (*TaWAK2A-800*-QF: 5′-GGTATCGTGCTACTGGAGCTC-3′, *TaWAK2A-800*-QR: 5′-CTCCCATGGTAGGC CTGTTA-3′), *TaRLCK1B* [40], *TaCERK1* (*TaCERK1*-QF: 5′-CGGAGCAGATGGAC ACACTT-3′, *TaCERK1*-QR: 5′-TGAGTGGCCGATGATGTCAC-3′), *TaMPK3* [41], *Defensin* [41], *Chitinase 3* [37], and *β-1,3-Glucanase* [37] in wheat were measured and analyzed using RT-PCR or qRT-PCR. Following the previous method [37], the qRT-PCR reaction was performed by using a SYBR Premix Ex Taq kit (TaKaRa, Otsu, Japan), in volumes of 20 μL, on an ABI 7500 RT-PCR system (Applied Biosystems, Waltham, MA, USA). The 2^−ΔΔCT^ method [42] was used to calculate the relative transcription level of the target genes (internal reference: *TaActin*). Three independent replications were performed in each test.

## 5. Conclusions

*TaWAK2A-800* was expressed to a higher extent in the *R. cerealis*-resistant wheat accessions than in the susceptible wheat ones. The gene transcript abundance is induced after *F. graminearum* infection and chitin treatment. The expressed *TaWAK2A-800* is required for the resistance of wheat to *R. cerealis* and *F. graminearum* by promoting the expression of *TaCERK1*, *TaRLCK1B*, *TaMPK3*, *Defensin*, *Chitinase 3*, and *β-1,3-Glucanase*. This work reveals a novel role for the wheat *TaWAK2A-800* gene in wheat plant immunity against pathogens. *TaWAK2A-800* is a potentially important gene for improving the broad-spectrum resistance of wheat to *R. cerealis* and *F. graminearum.*

## Figures and Tables

**Table 1:** The VIGS fragment was analyzed by Si-Fi software.

Target Genes	Total siRNA Hits	Efficient siRNA Hits
TraesCS2A02G071800.1	71	35
TraesCS2D02G070600.1	50	22

## Data Availability

The data presented in this study are available on request from the corresponding author.

## Supplementary Materials

The following are available online at https://www.mdpi.com/article/10.3390/ijms222111493/s1.
